# In Vitro Study of the Recruitment and Expansion of Mesenchymal Stem Cells at the Interface of a Cu-Doped PCL-Bioglass Scaffold

**DOI:** 10.3390/biomimetics7010019

**Published:** 2022-01-21

**Authors:** Behnaz Malekahmadi, Vahid Esfahanian, Fatemeh Ejeian, Maziar Ebrahimi Dastgurdi, Maria Agheb, Faranak Kaveian, Mohammad Rafienia, Mohammad Hossein Nasr-Esfahani

**Affiliations:** 1 Isfahan (Khorasgan) Branch, Dental School, Islamic Azad University, Isfahan 8155139998, Iran; behnaz.malekahmadi@yahoo.com; 2 Cell Science Research Center, Department of Animal Biotechnology, Royan Institute for Biotechnology, ACECR, Isfahan 8159358686, Iran; fatemeh.eje@gmail.com; 3 Isfahan (Khorasgan) Branch, Department of Periodontics, Dental School, Islamic Azad University, Isfahan 8155139998, Iran; 4 Private Practitioner (Endodontist), Toronto, ON L3T0C9, Canada; ebrahimimd@gmail.com; 5 Department of Biomaterials, Tissue Engineering and Nanotechnology, School of Advanced Technologies in Medicine, Isfahan University of Medical Sciences, Isfahan 8174673461, Iran; m_agheb@yahoo.com; 6 Biosensor Research Center, Isfahan University of Medical Science, Isfahan 8174673461, Iran; faranak58@gmail.com (F.K.); m_rafienia@med.mui.ac.ir (M.R.)

**Keywords:** GBR barrier membrane, cell adhesion, bioglass, mesenchymal stem cells, proliferation

## Abstract

Developing new barrier membranes with improved biomechanical characteristics has acquired much interest owing to their crucial role in the field of periodontal tissue regeneration. In this regard, we enriched the electrospun polycaprolactone (PCL)/gelatin (Gel) membranes by adding bioglass (BG) or Cu-doped bioglass (CuBG) and examined their cellular adhesion and proliferation potential in the presence of alveolar bone marrow-derived mesenchymal stem cells (aBMSCs). The membranes were fabricated and characterized using mechanical strength, SEM, FTIR, EDX, and ICP assay. Besides, aBMSCs were isolated, characterized, and seeded with a density of 35,000 cells in each experimental group. Next, the cellular morphology, cell adhesion capacity, proliferation rate, and membrane antibacterial activity were assessed. The results displayed a significant improvement in the wettability, pore size, and Young’s modulus of the PCL membrane following the incorporation of gelatin and CuBG particles. Moreover, all scaffolds exhibited reasonable biocompatibility and bioactivity in physiological conditions. Although the PCL/Gel/CuBG membrane revealed the lowest primary cell attachment, cells were grown properly and reached the confluent state after seven days. In conclusion, we found a reasonable level of attachment and proliferation of aBMSCs on all modified membranes. Meanwhile, a trace amount of Cu provided superiority for PCL/Gel/CuBG in periodontal tissue regeneration.

## 1. Introduction

Alveolar bone resorption is propounded as a severe health problem in the modern world affecting the quality of life, especially among aged populations [1]. It may be caused by periodontal disease, impaired bone metabolism, dietary deficiencies, or tooth loss and trauma. In this context, guided tissue/bone regeneration (GTR/GBR) is suggested as a promising alternative to autologous bone grafting techniques to reconstruct lost periodontal tissues [2,3,4]. This method is independent of extra invasive and harmful surgeries used in conventional procedures to harvest autologous iliac bone grafts. Alveolar bone marrow mesenchymal stem cells (aBMSCs) are the ideal cell source for use in GTR/GBR approaches because of their central role in natural bone repair via recruitment and homing to the defect sites. These cells can be easily obtained at the time of routine dental surgeries, such as dental implant treatment and third molar extraction. Based on their neural crest origin, aBMSCs show higher proliferation and osteogenic differentiation capacity and lower chondrogenic/adipogenic potential in comparison to iliac-derived cells [5].

Membranes are the major players of the standard GTR/GBR approaches by undertaking a barrier role and preventing the migration of cells from soft tissue to the defect area. Ideal GBR membranes not only have the remarkable ability to support cellular attachment and proliferation, but can also induce osteogenic differentiation. Namely, GBR membranes based on resorbable synthetic polymers have received major attention in periodontal regeneration approaches with respect to their safe healing, biocompatibility, and effective mechanical/physical properties [1,6]. Among all, poly (lactic acid) (PLA), poly (glycolic acid) (PGA), poly (e-caprolactone) (PCL), poly (hydroxyl butyric acid) (PHB), poly (hydroxyl valeric acid) (PHV), and their copolymers have been used frequently. In fact, using non-resorbable membranes is accompanied by additional surgery, causing discomfort to the patients. Hence, a growing number of studies have focused on designing a new generation GTR/GBR membranes with improved characteristics, e.g., low rigidity, shape-forming ability, degradability, and drug-encapsulating capacity [2,7,8]. Due to their easy manufacturing, suitable biocompatibility, and notable mechanical properties, PCL-based scaffolds have been widely used in biomedical applications. The specific hydrophobic and semi-crystalline structure of PCL caused slow degradation and resorption rates. This provides a good choice for approaches associated with long-term healing, e.g., bone, tendon, cartilage, and blood vessels regeneration. However, various modification methods have been applied for modulating its degradation rate and improving the biological responses to PCL substrates [2,9].

In contrast to PCL, gelatin (Gel) presented a relatively high degradation rate besides having integrin-binding sites and commercial availability at low cost. It is a well-known natural biomaterial produced from the hydrolysis of collagens under controlled enzymatic conditions. PCL/Gel composite provides good mechanical, physical, and chemical properties applied in cartilage regeneration, nerve engineering, wound dressing, cardiac tissue engineering, and muscle tissue engineering [6,8,10]. Three-dimensional (3D) nanofibrous scaffolds made of PCL/Gel composite presented outstanding potential to recapitulate the bone extracellular matrix (ECM) and improve the specific surface area of bioengineered tissue constructs [11].

In the context of bone tissue engineering, some kinds of inorganic constituents like bioactive glasses have received significant attention because of their high osteo-inductive properties. Indeed, ions released from the glass result in biological reactions at the cellular and tissue level [2,3,8,12]. Moreover, it is evident that doping bioactive glass compositions with various metallic elements (such as Zn, Sr, Cu, Mg, etc.) could provide additional advantages for therapeutic applications. Among different metal ions, a trace amount of copper (Cu^2+^) seems to have a positive impact not only on osteoblast attachment, proliferation, and osteogenesis, but also on angiogenesis and antibacterial activity. Angiogenesis has a special impact on vascularization through the grafted tissues and provides a suitable substrate for bone repair [12,13,14,15].

Here, we assessed the attachment and proliferation potential of aBMSCs on the surface of PCL/Gel/CuBG nanofibrous scaffolds prepared by electrospinning in comparison to PCL/Gel/BG and PCL/Gel scaffolds.

## 2. Experimentals

### 2.1. Isolation and Culture of aBMSCs

**Figure 1:**
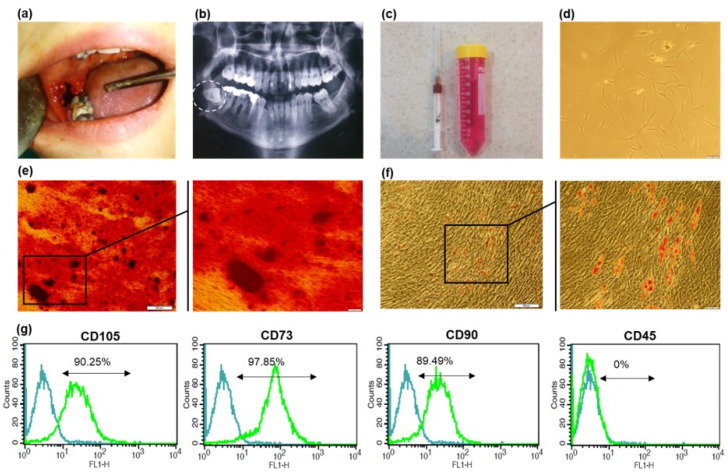
Isolation and characterization of a BMSCs. (**a**) Surgical extraction of impacted wisdom tooth (**b**) Panoramic radiography (**c**) Aspirated bone marrow in a heparinized syringe and alveolar bone particles in cell culture solution (**d**) Primary culture (P0) of aBMSCs with typical fibroblastic morphology (scale bar 100 µm) (**e**) Alizarin Red staining and (**f**) Oil Red staining of aBMSCs, following three weeks of osteogenic and adipogenic differentiation induction (×4, ×20 magnification) (**g**) The flow cytometric plots for specific MSC surface markers (CD105, CD90 and CD73) and CD45 as a hematopoietic surface marker. Isotype-matched antibodies were used as the internal control for all markers.

Alveolar bone marrow stem cells were obtained from three healthy patients undergoing surgical extraction of the impacted third molar. Written informed consent was obtained from the patients in accordance with the Helsinki Declaration. This study was approved by the ethics committee of Isfahan University of Medical Sciences and Islamic Azad University (Isfahan (Khorasgan) Branch), Faculty of Dentistry (Code: IR.MUI.REC.1395.4.30). The standard procedure established by Mason et al. for the safe isolation of aBMSCs from human patients has been applied with a minor modification [16]. Briefly, bone marrow samples were collected from the two distinct surgical area: (i) alveolar bone particles extracted to reach the impacted tooth and (ii) bone marrow aspirated from opens socket. Following the extraction of the tooth, an 18-gauge long needle was applied in connection to a heparinized syringe (Figure 1a–c). Both specimens were placed on the 40-micrometer nylon mesh, immersed in Dulbecco’s phosphate buffered saline (DPBS; Gibco, Paisley, UK), and centrifuged twice at 600× *g* for 10 min. The aspirated bone marrows (approximately 1–1.5 mL) were pooled from three patients and combined with the collected cells from the scraped bone. Finally, cells (around 6 × 10^5^ cells) were cultured in an alpha modification of minimum essential medium (α.MEM; Gibco, Paisley, UK) supplemented with 20% (*v*/*v*) fetal bovine serum (FBS; Gibco, Paisley, UK), 2% (*v*/*v*) penicillin/streptomycin (Pen/Str; Gibco, Paisley, UK), 2% (*v*/*v*) amphotericin (Sigma, Munich, Germany), 2% (*v*/*v*) gentamicin (Sigma, Munich, Germany), 1% (*v*/*v*) l-glutamine (L-Glu; Gibco, Paisley, UK), and 1% (*v*/*v*) nonessential amino acid (NEAA; Gibco, Paisley, UK) and were incubated in 37 °C humidified at 5% CO_2_.

After 24 h, culture plates were gently washed with DPBS to eliminate debris and non-adherent cells. The growth medium was refreshed twice a week and cells were passaged at around 90% confluency using trypLE solution (Gibco, Paisley, UK). According to our previous report on the isolation of stem cells from exfoliated deciduous teeth (SHEDs) [17], after three passages, the growth medium was changed to Dulbecco’s modified eagle medium (DMEM; Gibco, Paisley, UK) supplemented with 10% FBS, 1%Pen/Str, 1% amphotericin, 1% gentamicin, and 1% L-glutamine. The cells within the 4–8 passages were used for further experiments.

### 2.2. Characterization of Isolated Cells

According to the minimal criteria established by the international society of cellular therapy (ISCT) for MSCs, morphological features and the expression of specific surface markers were assessed. [18]. For the immunophenotypic analysis of cells, the expression level of main MSC markers (CD105, CD90, CD73) was assessed in combination with CD45 as a hematopoietic stem cell marker. The cell suspensions were initially stained with specific primary antibodies (all from Merck Millipore, Billerica, MA, USA) and then labeled with appropriate secondary antibodies. The assessment was performed using flow cytometry (Becton Dickinson, San Jose, CA, USA). To evaluate the proliferation rate of cells in normal culture condition, population doubling time (PDT) was calculated at each passage between passages 2 and 6 using the following formula:

(1) PDT=duration of culture in days/(log Nh− log Ni)/log2

where Nh resembles the number of harvested cells and Ni refers to the number of initially plated cells [16].

For the evaluation of multilineage differentiation capacity of isolated cells, they were induced to differentiate to osteoblast and adipocytes by applying the specific medium, as previously reported for dental pulp stem cells [19]. After three weeks, the osteogenesis and abiogenesis potential were measured, respectively, through staining with Alizarin Red and Oil Red according to previously established methods [20].

### 2.3. Scaffolds Materials and Preparation

For pure PCL samples, PCL (Mn = 80,000, Sigma, Munich, Germany) dissolved in 1,1,1,3,3,3-Hexafluoro-2-propanol (HFP, Sigma, Munich, Germany) to reach a 10 wt.% solution. To prepare PCL/Gel composite, PCL and gelatin (Mn = 250,000, Merck) were dissolved in HFP in the 50:50 weight ratio to obtain a ten wt.% solution. The mixture was stirred for 8 h at room temperature to obtain a clear solution. For PCL/Gel/BG and PCL/Gel/CuBG nanocomposites, after preparing 9 wt.% PCL/gelatin clear solution, 1 wt.% of the 45S5 bioglass^®^ (BG < 100 nm, Nikceram-Razi, Isfahan, Iran) and Cu-doped 45S5 bioglass (CuBG, <150 nm, Nikceram-Razi) were added separately to the solutions to get the final concentration of 10 wt.%. The mixture was stirred for an additional 4 h to gain a homogeneous solution. The exact composition of bioglass and Cu-bioglass is shown in Table 1.

Electrospinning was carried out using a 1 mL syringe equipped with a 19-gauge flat-tip needle connected to the positive high voltage power supply, which applied 12–13 kV. The collector was covered with a 15 × 15 cm^2^ aluminum sheet and placed 15 cm far from the needle tip. The constant flow rate of 1 mL/h was applied to fabricate all membranes. The precise classification and composition of samples are identified in Table 2. After collecting the samples, all of them dried were overnight under vacuum at room temperature to let the solvent evaporate entirely from the fibers.

### 2.4. Scaffold Characterization

#### 2.4.1. Morphology Analysis

The morphological feature of electrospun membranes was observed using a scanning electron microscope (SEM, Hitachi S4160). Prior to imaging, samples were coated with a thin layer of gold to produce a conductive surface. The fibers diameter and average surface pore size were calculated by using ImageJ software, and the porosity of the scaffolds in the first layer was measured using the MATLAB program.

#### 2.4.2. Mechanical Characterization

Mechanical characteristics of samples were assessed with an Instron 5566 universal testing machine at room temperature using a 10 N load cell with 10 mm/min cross-head speed, according to the ASTM D638 standard test method. All samples were prepared in 5 × 30 mm^2^ dimensions. The data concerning tensile stress, modulus, and strain at break were the average of five specimens.

#### 2.4.3. Hydrophilicity

The water contact angle on the surfaces of the scaffolds was measured 30 s after drop falling as the indicator of hydrophilicity, using the contact angle measuring machine (CA-X Contact Angle Meter from Kyowa Interface Science Co., Niiza, Japan) equipped with a CCD camera.

#### 2.4.4. FTIR Assessment

The investigation of chemical reactions and determination of functional groups was carried out by Fourier transform infrared spectroscopy (FTIR) (FTIR; IFS-66 V/S, Bruker, Ettlingen, Germany) over a wavenumber range between 400 and 4000 cm^−1^ at room temperature. Hence, 1 mg of the sample was ground to powder form, then mixed with 300 mg of KBr and palletized under vacuum.

#### 2.4.5. Mineralization Assay

In order to assess the in vitro bioactivity of composite scaffolds, the nucleation level of hydroxyapatite was analyzed utilizing SEM (Hitachi S4160) imaging in combination with EDX analysis, which was performed using the EDX accessory on the SEM instrument. For each sample, three specimens (10 × 10 mm^2^) were soaked in 10 mL simulated body fluid (SBF) (regarding the kokubo formulation) [21], in the vertical position for 28 days at 37 °C. Afterward, samples were rinsed with pure water and vacuum dried at room temperature to prepare for SEM investigation [22].

#### 2.4.6. Evaluation of Cu Ion Release

Ion release (Cu^2+^) from scaffolds was quantized using an inductively coupled plasma optical emission spectrophotometer (ICP-OES, Thermo Scientific iCAP 6500, Waltham, MA, USA). The amount of Cu^2+^ released from the PCL/CuBG nanofiber scaffold was determined by immersing the (4 × 4 cm^2^, 0.2 g) of samples into 10 mL of PBS at different time points.

#### 2.4.7. Antibacterial Property of the Scaffolds

The diffusion method was selected as the screening assay to verify the antibacterial efficacy of different scaffolds. The antibacterial activity of sterilized nanofibrous membranes (5 mm^2^) was evaluated against *Porphyromonas gingivalis* purchased from the Pasteur Institute of Tehran, Iran. The agar surface was inoculated using a swab dipped in a cell suspension of a 0.5 McFarland turbidity standard, approximately corresponding to 1 × 10^8^ colony forming unit per mL (CFU/mL). All the scaffolds were placed on *Porphyromonas gingivalis* inoculated agar plate and incubated at 37 °C for 24 h. Finally, the inhibition zone diameters were measured.

### 2.5. Cell Attachment and Proliferation Assessment

To study the level of cell attachment and proliferation on the prepared membranes, cell viability was measured using the MTS-based Cell Titer 96^®^ assay (Promega, Madison, WI, USA) according to the manufacturer’s instruction [23]. The optimal number of aBMSCs was determined as 3.5 × 10^4^ cell/cm^2^, based on a series of cell concentrations on the PCL/Gel as the control scaffold. Cell adhesion was assessed after 4 h incubation, and proliferation was determined after 1, 3, 5, and 7 days under normal culture conditions. For the MTS assay, cells were treated for 3 h with MTS/PMS working solution, and the formazan absorbance was measured at 450 nm. The tissue culture plate was also examined as the positive control for attachment and proliferation assays.

### 2.6. SEM Analysis

The cellular recruitment as well as morphological features of adhered cells were assessed with the aid of SEM (Philips XL30, Philips, The Netherland). After 1 day, samples were fixed with 2.5% glutaraldehyde (Sigma, Munich, Germany) for 1 h and dehydrated through increasing grades of ethanol (25%, 50%, 75%, 90%, 100%) for 15 min at each step [23]. Then, specimens were coated with a 10-nm gold layer and observed by electron microscopy. All cell-free scaffolds also were assessed as the control groups.

### 2.7. Statistical Analysis

All quantitative data are reported as mean value ±SE of at least three independent experiments, and analysis was performed using one-way ANOVA through SPSS 16.0 software 25. *p* values < 0.05 were considered statistically significant for all experiments.

## 3. Result

### 3.1. Stem Cell Characterization

Primary MSC colonies appeared after 10 days post-primary plating (Figure 1d). Adhered cells presented fibroblast-like morphology in the early days of culture and kept their typical spindle morphology of BMSCs after the first passages.

Based on our results, the population doubling time (PDT) of these cells was calculated as 52–54 h through passage 3–6, which is within the normal range for mesenchymal stem cells [24].

The immune-phenotypic analysis of isolate cells revealed high expression of common mesenchymal stem cell markers (CD105 (90.25%), CD90 (89.49%), and CD73 (97.85%)). The cells were also negative for CD45 as a hematopoietic marker. Figure 1g shows the flow cytometric representative histogram of each marker. Figure 1e,f demonstrates the in vitro multilineage differentiation potential of the cultured cells as a critical character for MSC identification. Specific Alizarin red staining of mineral depositions shows a relatively high tendency of these cells towards osteogenic differentiation. Cells that undergo adipogenic differentiation also presented remarkable oil droplets, which were stained in red using Oil red.

### 3.2. Membrane Characterization

**Figure 2:**
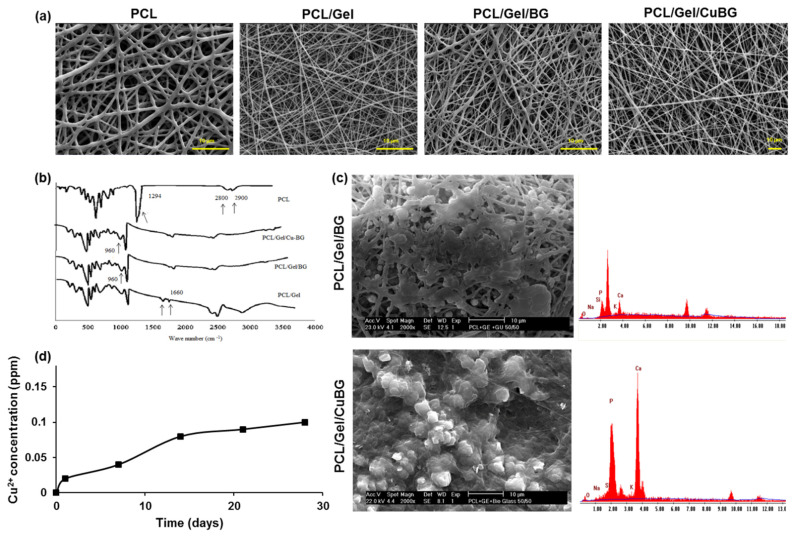
Membrane characterization. (**a**) SEM images of the electrospun nanofibrous membranes. (**b**) FTIR spectra of scaffolds (**c**) SEM images of membranes after 28 days soaking in SBF (**d**) Cu^2+^ ions released in water from the PCL/Gel/BG, PCL/Gel/CuBG hybrid nanofiber scaffolds with spectto incubation time.

SEM images of nanofibrous scaffolds are presented in Figure 2a. Fibers are bead-free, and powders are homogeneously dispersed in the polymer matrix. Pore distribution is homogeneous in all samples. Physical properties of the nanocomposite scaffolds are reported in the Table 3. PCL fiber thickness was three times higher than other samples, primarily due to its non-polar nature. The incorporation of gelatin with PCL caused thinner fiber diameter scaffolds since gelatin can ionize in the solvent to carboxylic and amine functional groups. Therefore, the charge density on the surface of polymer jet is higher compared to the pure PCL. Higher conductivity in the polymer solution supports the low fiber diameter [25]. The results revealed that the incorporation of BG and CuBG increased the fiber diameter of the electrospun scaffolds in comparison with PCL/Gel samples. This is due to an increase in the total concentration of the electrospun solution [26].

The mechanical properties of PCL, PCL/Gel, PCL/Gel/BG, and PCL/Gel/CuBG nanofibers are given in Table 4. The average tensile strength of PCL and PCL/Gel nanofibers were 3.12 ± 0.021 MPa and 2.36 ± 0.064 MPa with strain at break of 176% and 85%, whereas PCL/Gel/BG and PCL/Gel/CuBG nanofibers had a tensile strength of 3.82 ± 0.036 MPa and 3.54 ± 0.045 MPa with strain at break of 76% and 81%.

The existence of gelatin and bioglass in the scaffolds leads to an increase in the hydrophilicity of the composite due to the potential of amide groups for hydrogen bonding with OH groups in the aqueous environment. The low hydrophilicity of PCL is not favorable for cell growth [27]. The contact angle was reduced more than 3.5 times in PCL (105° ± 6) in comparison with PCL/Gel (28° ± 3) samples. BG and CuBG increased the hydrophilicity of the matrix and made it completely wettable. All samples had porosity above 80%, which is in favor of a suitable scaffold. The pore size in all nanocomposite samples is above 10 (μm), which is in favor of growth and cell migration or infiltration [28].

Pure PCL showed higher strength among other scaffolds. Gelatin addition decreased strain and tensile strength in all composite specimens due to the weak physical properties of the gelatin [29]. The combination of bioceramic fillers in polymeric scaffolds in most cases enhanced mechanical properties in comparison with the pure polymer. However, it could alter the biodegradation and cytotoxicity of the composite scaffold [30].

### 3.3. FTIR Analysis

Figure 2b shows the FTIR spectra of the scaffolds. The BG characteristic peaks related to Si-O-Si vibrating bonds and Si-O appeared in 960 and 920 cm^−1^ regions, respectively. Peaks at 2800–2900 cm^−1^ revealed -CH2- and -CH- bonds, and a peak around 1700 cm^−1^ is assigned to carbonyl stretching bond (C=O) in PCL. A strong peak at 1294 cm^−1^ is related to C-C and C-O stretching bonds in the PCL backbone [30]. The presence of gelatin characteristic peaks at 1660 cm^−1^ for C=O stretching bond for amide I, and N-H bend and C-N stretching at 1540 cm^−1^ for amide II, in PCL/Gel composites showed the appropriate incorporation of gelatin and PCL [29].

Peaks in the 3300–3500 cm^−1^ range are related to the O-H bond. Amide groups in gelatin are able to form hydrogen bonds with water molecules. Thus, hydrophilic functional groups in the gelatin molecule could increase the hydrophilicity of PCL composite nanofibers [31].

### 3.4. Mineralization Assay

As shown in Figure 2c, the nucleation of hydroxyapatite is obvious on the fibers after soaking in SBF at 37 °C. The EDX spectra of the hybrid electrospun fiber mat, shown in Figure 2c, revealed a homogeneous distribution of carbon, calcium, silicon, and phosphorus atoms. The results from SEM and EDX analysis revealed the development of the apatite layer on the surfaces and pores of scaffolds after immersion in SBF solution.

### 3.5. Copper Release

Figure 2d depicts the results obtained from the Cu^2+^ ions release experiment. It is observed that Cu^2+^ ions from the PCL/Gel/CuBG nanofiber scaffold are released in a sustained manner.

The amount of Cu^2+^ ions released from the PCL/Gel/CuBG nanofiber scaffold reached 0.08 ppm after 14 days of incubation in SBF.

### 3.6. Antibacterial Activity

**Figure 3:**
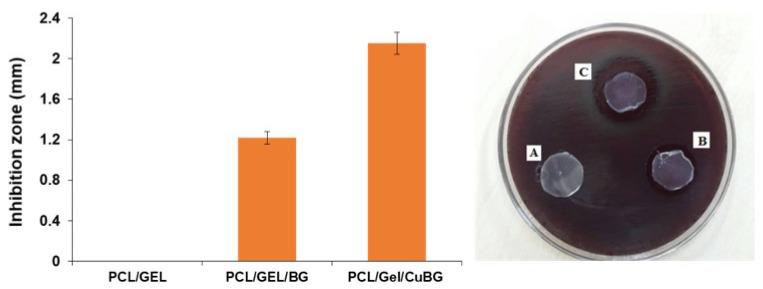
Antibacterial activity of the membrane. The inhibition zones of PCL/Gel, PCL/Gel/BG, PCL/Gel/CuBG scaffolds against *porphyromonas gingivalis* after 24 h incubation are shown in A, B, and C, respectively.

Antibacterial analysis against *Porphyromonas gingivalis* revealed a moderate inhibition zone size around PCL/Gel/BG (1.22 ± 0.18 mm), which was increased significantly in the case of PCL/Gel/CuBG (2.15 ± 0.25 mm). However, PCL/Gel membrane disc did not exhibit any sign of antibacterial activity (Figure 3).

### 3.7. Cell Attachment and Proliferation Assessment

**Figure 4:**
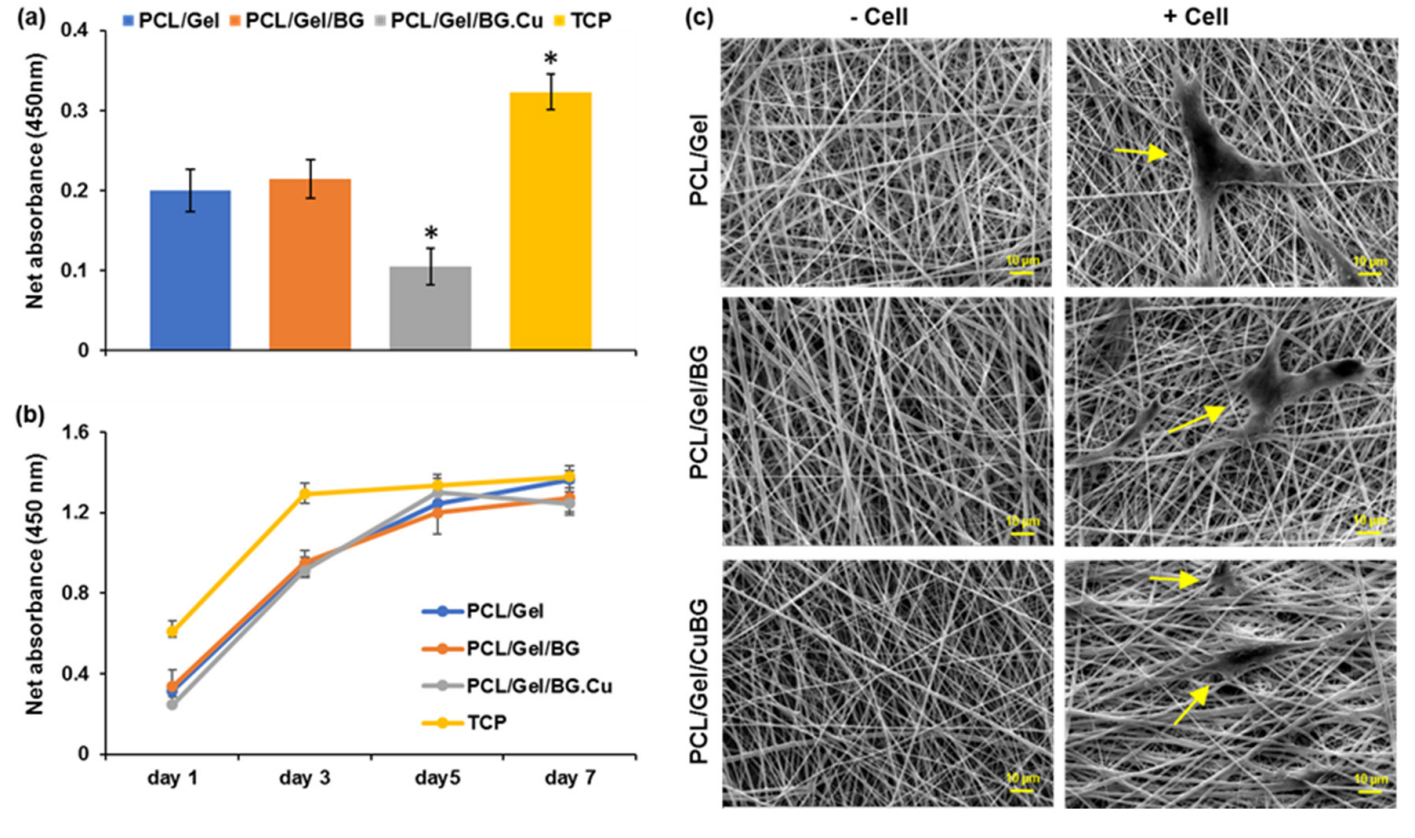
Cellular attachment and proliferation on substrates (**a**) The primary attachment of aBMSCs on the surface of PCL/Gel and PCL/Gel/BG and PCL/Gel/CuBG, as well as TCP as the positive control, measured by MTS assay (* *p*-value < 0.05) (**b**) The proliferation rate of aBMCSs on the surface of scaffolds and TCP at different time points during a week. All monitored absorbance were normalized to cell-free control of each membrane (**c**) SEM images of three scaffolds after one day. aBMSCs are shown with arrows.

To quantify the initial attachment capacity and proliferation rate of aBMSCs on the scaffolds, the MTS assay was performed. According to the cell number titration, the optimal cell seeding density for attachment analysis was determined as 18,600 cells/cm^2^ (data have not been shown). The initial cell attachment analysis after four hours revealed a significantly higher tendency of aBMSCs to tissue culture plate (TCP) in comparison to all three PCL-based scaffolds (*p*-value < 0.05). Among evaluated membranes, we found the lowest level of primary cell attachment on PCL/Gel CuBG membrane (Figure 4a), while an increased number of cells was observed on PCL/Gel and PCL/Gel/BG scaffolds (*p*-value < 0.05) after four hours. The rate of cell proliferation through seven days for all groups is presented in Figure 4b. All membranes show a similar trend of cell proliferation during test time. Although significantly lower amounts of cells were detected on membranes until day 5, they almost reached the plateau state of the control TCP group on day 7 (Figure 4b).

### 3.8. Monitoring the Morphological Feature of Cells on Membranes

It is obvious in Figure 4c that smooth electrospun nanofibers were arrayed randomly with a relatively similar diameter in all membranes. Cultured aBMSCs are expanded on the surface, and their spindle shape morphology is clearly detectable.

## 4. Discussion

Tissue engineering strategies for bone regeneration typically rely on the use of an artificial extracellular matrix (scaffold), stem cells, and inducing factors that promote cell recruitment and osteo-differentiation [32]. In this study, we focused on PCL-based membranes that have been widely used in craniofacial areas. Hence, we applied aBMSCs having notable proliferation potential and a critical role in natural bone repair [5,33]. Due to their accessibility and migration capacity, aBMSCs are considered as the best candidate for cell therapy strategies in craniofacial defects. In the current research, PCL/Gel, PCL/Gel/BG, and PCL/Gel/CuBG scaffolds were successfully prepared via the electrospinning process, and their potential as the GBR barrier membrane has been examined.

Incorporating gelatin into PCL caused a thinner fiber diameter in the scaffolds, since gelatin could be ionized in the solvent and caused higher charge density on the surface of polymer jet compared to the pure PCL. This is in line with a previous report by Son et al. that the higher conductivity in polymer solution could lead to a lower fiber diameter [25]. On the other hand, adding BG and CuBG could result in a higher fiber diameter, because of the higher concentration of the electrospun solutions [26]. When the fiber’s diameters are similar to or larger than the cells, the pseudopodiums cannot cover the fibers. However, cells can wrap around the small fibers and become like a bridge between them. Moreover, it is shown that the large specific surface area of thin fibers is associated with the greater adsorption of biological components and superiority for supporting cell proliferation [34]. A previous study by Lyu et al. showed that the maximum osteogenic differentiation was achieved on the scaffolds with the fiber diameter ranging between 300 and 1300 nm [35]. We found that all the scaffolds were in the appropriate range of fiber diameter (400–600 nm) for the critical activity of the cells. The best efficiency was observed using the PCL/Gel/CuGP membrane, which is likely attributed to its suitable porosity, hydrophilicity, and mechanical characteristics.

Given the importance of good mechanical properties for the widespread clinical application of GBR barrier membranes, several studies have been conducted to develop membranes with optimal mechanical/physical characteristics. Mainly, GBR membranes should provide sufficient stability and tensile strength to maintain the space of bony defects. However, they are required to have adequate plasticity to easily adapt to the shape [36]. Moreover, the intermediate level of porosity would be beneficial for cell proliferation and nutrient availability along with the inhibition of cell infiltration from soft tissue to the defect [37]. In this regard, the mechanical properties of fabricated membranes are consistent with the particular requirements defined by Ren et al. [29] and Wu et al. [38] for GBR barrier membranes. In fact, a combination of bio-ceramic dopant is usually used for improving the mechanical characteristics of polymeric scaffolds. This modification approach would be advantageous for improving the biodegradation rate and cytotoxicity level of membranes [30]. According to Table 4, PCL/Gel/BG and PCL/Gel/CuBG scaffolds showed higher tensile strength and better flexibility than PCL and PCL/Gel nanofibers, which could be attributed to the formation of secondary bonds between BG particles and the matrix [39]. Likewise, Shirani et al. reported that using electrospun nanocomposite scaffolds is accompanied with improved mechanical properties and higher bone-regeneration potential compared to using the PCL scaffold [40,41]. Besides, all fabricated membranes present appropriate porosity in the first layer, which in turn provides a large surface area for cell attachment.

Meeting the ideal criteria of GTR/GBR membranes, scaffolds are required to possess bioactivity and promote osteo-regeneration. EDX analysis showed a suitable Ca/P ratio (approximately 1.67) in PCL/Gel/CuBG samples, which corresponded to the nonstoichiometric biological apatite. It is supposed that the Cu-doped membrane had more reactivity in SBF due to ion release from the scaffold. Furthermore, the results showed that incorporating Cu^2+^ ions into the basic 45S5-bioglasses gives them antibacterial property against *Porphyromonas gingivalis*. Since bacterial infections are unavoidable, especially in large bone defects, the antibacterial activity could be considered as an influential advantage for any bioactive scaffolds [42].

The wettability is defined as a critical cue of substrates with a profound impact on the bioactivity of scaffolds. Our findings verified that the presence of gelatin and bioglass improved the hydrophilicity of the membrane due to the tendency of amide groups towards hydrogen bonding with OH in the aqueous environment. Based on the literature, mesenchymal stem cells revealed the highest level of cellular adhesion and proliferation on the substrates with moderate wettability (contact angle: 40–70°) [33,35]. This fact is in line with the moderate cellular attachment on bioglass-supplemented substrates exhibited relatively low water contact angle (PCL/Gel/BG: 14 ± 3° and PCL/Gel/CuBG: 10 ± 2°). On the other hand, the hydrophobic nature of the PCL membrane could prevent effective cellular adhesion and expansion and showed a negative impact on its osteo- inductive function.

Several lines of evidence showed that the high concentrations of Cu^2+^ could induce hypoxia response and cell death in different plant and animal tissues [12,43,44]. This is while we found that the slow release of copper from PCL/Gel/CuBG substrate not only has no adverse effect in the long-term, but can also provide a unique opportunity for promoting bone regeneration. In particular, Weng et al. observed a positive influence of 1% Cu-doped BG electrospun fibers on different human cell lines with no significant cytotoxicity [45]. Moreover, Seth et al. reported copper cytotoxicity against Hep G2 cells in concentrations over 100 μM [46] and the half-maximal inhibitory concentration (IC50) of CuO-Nanoparticle on lymphocytes was determined as 382 μM by Assadian et al. [47].

Primary cellular attachment analysis on the first day shows that the cell recruitment on the TCP is still higher than others. Moreover, the proliferation rate on PCL/Gel and PCL/Gel/BG was significantly higher than PCL/Gel/CuBG scaffold with no significant increase compared to the primary adhesion. Regarding the previously mentioned doubling time (52–54 h), cells need at least 52 h to reach double numbers. After three days, there was a significant difference only between TCP and other groups that are subordinate to the higher cells’ attachment on the surface of TCP compared to other groups. Until day 3, the cells on the scaffolds are in the growth logarithmic phase and follow a similar proliferation pattern, which indicates the non-toxicity of the scaffolds on natural cell growth and proliferation. In the case of the TCP group, it seems that aBMSCs have reached the plateau state after three days. On day 5, aBMSCs on the surface of scaffolds are still in optimum growth condition, and there is only a significant difference between TCP and PCL/Gel/BG groups (*p* > 0.05). After five days, results show a higher cell proliferation rate on the surface of the PCL/Gel/CuBG scaffold, which reaches control confluency, despite there being less primary adhesion. Our finding is in agreement with the results obtained by Haider et al. on PGA/Cu nanofibrous scaffolds [48].

It is worth noting that the cell proliferation curve of the PCL/Gel scaffold still increases until day seven, while the slight decline in MTS absorption on PCL/Gel/BG and PCL/Gel/CuBG scaffolds may reflect decreasing cell metabolic activity that could be related to differentiation induction by BG and Cu [33,49].

## 5. Conclusions

In conclusion, these findings indicated that all three scaffolds revealed a reasonable tendency for cellular attachment and proliferation. However, adding CuBG to PCL/Gel scaffold does not only have any toxic effect on aBMSCs in the long-term, but also caused a sensible antibacterial effect to control infection in the defect areas. Therefore, these findings underscore the potential of the PCL/Gel/CuBG scaffold as a promising GBR barrier membrane with remarkable osteoconductive and antibacterial potential for use in GTR/GBR approaches to periodontal regeneration strategies.

## Figures and Tables

**Table 1:** Bioglass and Cu-bioglass composition.

Glass	Composition (wt.%)				
	SiO_2_	Na_2_O	CaO	P_2_O_5_	CuO
Bioglass 45S5	45	24.5	24.5	6	-
Cu-bioglass	45	24.5	23.5	6	1

**Table 2:** Samples compositions.

Sample	Polymers	Weight Ratio (%)
1	PCL	100
2	PCL/Gel	50/50
3	PCL/Gel/BG	50/50.1% BG
4	PCL/Gel/CuBG	50/50.1% CuBG

**Table 3:** Physical properties of nanofiber scaffolds.

Sample	Mean Fiber Diameter (nm)	First Layer Porosity (%)	Mean Pore Size (μm)	Contact Angle (°)
PCL	622.72 ± 93	85.08	7.85 ± 3	105 ± 6
PCL/Gel	407.56 ± 66	82.93	11.35 ± 5	28 ± 3
PCL/Gel/CuBG	495 ± 22	82.82	12.82 ± 8	10 ± 2
PCL/Gel/BG	486 ± 42	83.26	10.52 ± 6	14 ± 3

**Table 4:** Mechanical properties of PCl, PCl/Gel, PCl/Gel/BG and PCl/Gel/CuBG Scaffolds.

Sample	Tensile Strength (MPa)	Young’s Modulus (MPa)	Strain (%)
PCL	3.12 ± 0.021	1.14 ± 0.01	176 ± 23
PCL/Gel	2.36 ± 0.064	3.51 ± 0.03	85 ± 16
PCL/Gel/BG	3.82 ± 0.036	3.95 ± 0.02	76 ± 12
PCL/Gel/CuBG	3.54 ± 0.045	3.78 ± 0.02	81 ± 21

## Data Availability

Not applicable.
